# Exposure to natural pathogens reveals costly aphid response to fungi but not bacteria

**DOI:** 10.1002/ece3.892

**Published:** 2014-01-23

**Authors:** Seth M Barribeau, Benjamin J Parker, Nicole M Gerardo

**Affiliations:** Department of Biology, Emory University, O. Wayne Rollins Research Center1510 E. Clifton Rd. N.E., Atlanta, Georgia, 30322; Institute for Advanced Study Berlin (Wissenschaftskolleg zu Berlin)Wallotstrasse 19, 14193, Berlin, Germany; Experimental Ecology, Institute of Integrative Biology, ETH ZürichUniversitätstrasse 16, Zürich, 8092, Switzerland

**Keywords:** Ecological Immunology, host-pathogen interactions, *Acyrthosiphon pisum*, *Pandora neoaphidis*, immunity

## Abstract

Immune responses are costly, causing trade-offs between defense and other host life history traits. Aphids present a special system to explore the costs associated with immune activation since they are missing several humoral and cellular mechanisms thought important for microbial resistance, and it is unknown whether they have alternative, novel immune responses to deal with microbial threat. Here we expose pea aphids to an array of heat-killed natural pathogens, which should stimulate immune responses without pathogen virulence, and measure changes in life-history traits. We find significant reduction in lifetime fecundity upon exposure to two fungal pathogens, but not to two bacterial pathogens. This finding complements recent genomic and immunological studies indicating that pea aphids are missing mechanisms important for bacterial resistance, which may have important implications for how aphids interact with their beneficial bacterial symbionts. In general, recent exploration of the immune systems of non-model invertebrates has called into question the generality of our current picture of insect immunity. Our data highlight that taking an ecological approach and measuring life-history traits to a broad array of pathogens provides valuable information that can complement traditional approaches.

## Introduction

Invertebrates rely on innate immune mechanisms for protection against diverse parasitic organisms. Our current model of insect innate immunity relies heavily on knowledge from relatively few model organisms (e.g., *Drosophila melanogaster* (Lemaitre and Hoffmann 2007), *Anopheles gambiae* (Christophides et al. 2004)*, Tribolium castaneum* (Zou et al. 2007)). The immune systems of some insects, however, differ from these models of innate immunity (Evans et al. 2006; Smith et al. 2011a,b2011b), questioning the generality of our current picture of insect immunity. Assays of pea aphid (*Acyrthosiphon pisum*) immunity, using both genomic and experimental approaches, have found a reduced complement of conventional immune mechanisms. Pea aphids lack many presumably critical immune genes (e.g., bacterial recognition molecules [including Peptidoglycan recognition proteins] and much of the IMD [immunodeficiency] pathway [Gerardo et al. 2010]), have relatively few hemocytes (Laughton et al. 2011; Schmitz et al. 2012), have weakly functioning lysozymes (Altincicek et al. 2008), and have no detectable antimicrobial peptides via standard functional (Laughton et al. 2011) and proteomic assays (Gerardo et al. 2010). We expect that exploration of immune mechanisms across more diverse hosts, which is being facilitated by the declining costs of genome sequencing and thus of comparative genomics, will lead to a growing number of examples of organisms that do not fit the conventional models of immunity. In these situations, it is difficult to determine whether organisms are unable to respond to particular parasites or whether they are responding to parasite challenge using unknown mechanisms. Addressing these two possibilities will facilitate investigation of the evolution of host-microbe associations in many non-model systems of host-parasite coevolution and symbiosis.

Since all previous attempts to characterize pea aphid immune responses have relied on existing models of immunity we exploited the fact that mounting an immune response results in an energetic cost that can be measured in other traits, such as in reduced reproduction. Immune responses come at a high energetic cost, causing a trade-off between pathogen defense and other life history traits (Sheldon and Verhulst 1996; Rolff and Siva-Jothy 2003). Here we illustrate that by measuring life-history traits of organisms after pathogen exposure we can reveal immune responses that are recalcitrant to traditional approaches (Boughton et al. 2011). We expose aphids to several heat-killed natural aphid pathogens—two species of Gram-negative bacteria, a Gram-positive bacterium, and two species of aphid-specific entomopathogenic fungi—and measure fitness traits after exposure.

## Materials and Methods

### Pathogens

#### Fungal pathogens

*Zoophthora occidentalis* and *Pandora neoaphidis* are both aphid specific fungal entomopathogens. We cultured *Zoophthora occidentalis* in 100 mL potato dextrose broth shaking at room temperature for 2 days. We then passed the total culture through a vacuum filter and scraped the filtered fungal culture into 250 *μ*L Ringers solution. We cultured *Pandora neoaphidis* on plates of SDAEY (Papierok and Hajek 1997) for 14 days, and scraped 1 cm^2^ of fungal growth into 250 *μ*L of Ringer's solution. Previous work has shown that pea aphids are highly susceptible to these pathogens (Parker et al. 2013). As in previous studies (Vilcinskas and Götz 1999; Altincicek et al. 2008), we challenged aphids with heat-killed solutions (by autoclaving the exposure solutions at 121°C for 20 min) in order to assess the costs of immune activation without allowing the pathogens to establish an infection.

#### Bacterial pathogens

We isolated bacteria from laboratory stock aphids by crushing individual sick aphids in 500 *μ*L Carlson's solution and plating a portion of this solution onto Luria Broth (LB) plates, which were then cultured at 28°C overnight. We sequenced a portion of the 16s RNA gene (primers 27F: 5′-AGAGTTTGATYMTGGCTCAG, 1492R: 5′-TACCTTGTTAYGACTT) and identified the bacteria using the Ribosomal Database Project (Cole et al. 2009), NCBI BlastN and phylogenetic analyses (data not shown). Strain Ng5b is *Enterobacter* c.f. *cloacae*, strain n1324b is *Bacillus* c.f. *pumilus*, and strain s8d is *Serratia* c.f. *fonticola*.

To assess pathogenicity of the bacteria strains, we plated bacteria onto LB from glycerol stocks and grew them overnight at 30°C. We then picked multiple colonies and grew then to OD_600_ = 0.5. We stabbed six-day old aphids (line 5A0) with a minutin pin dipped into either sterile LB (control) or the live bacterial solution (Altincicek et al. 2011). In two experiments (first experiment: control sterile stab, Ng5b, s8d; second experiment: control sterile stab, Ng5b, n1324b), we stabbed 12 aphids per treatment sub-cuticularly into the ventral side of the abdomen and to one side of the midline to avoid rupturing the gut. Thirty minutes after stabbing, aphids were transferred from sterile Petri dishes to fava bean plants and monitored for survival.

As with the fungal elicitors, to measure the costs associated with mounting an immune response rather than the damage caused by infection, we exposed the insects to heat-killed pathogens. We cultured bacteria Ng5b and n1234b in LB overnight at 37°C, standardized a final volume of 250 *μ*L to OD_600_ = 0.5, spun each suspension at 2000× g for two minutes and resuspended the pellets in 250 *μ*L Ringers solution. To make a more concentrated solution of s8d, we followed the same procedure but resuspended a pellet from 2000 *μ*L bacterial solution (OD_600_ = 0.5) in 250 *μ*L Ringers solution. Finally, we heat-killed the bacteria by autoclaving the solutions at 121°C for 20 min.

### Cost of pathogen signal exposure

We maintained aphids asexually on fava bean (*Vicia faba*) plants in 16 h light: 8 h dark conditions at 20°C. We used aphid clones 5A0 (Oliver et al. 2003) and LSR1-01 (IAGC 2010), which are free of secondary, facultative symbionts but harbor the obligate bacterial symbiont, *Buchnera aphidicola*. Pea aphids produce two distinct phenotypic morphs, a dispersing winged morph and a sedentary unwinged morph. Immune costs are often context dependent, only appearing under energetically limiting conditions (e.g., Moret and Schmid-Hempel 2000). We therefore targeted costs of pathogen exposure in winged aphids because they have the additional energetic burden of producing wings and the associated musculature (Artacho et al. 2011). To induce the production of winged offspring, we exposed developing aphids to the alarm pheromone (E)-*β*-farnesene (EBF) (5 *μ*L of 1000 ng/*μ*L EBF every 48 h for 10 days). We then grew the offspring of these EBF-exposed aphids for 6 days, and exposed them to a suspension of heat killed pathogen by stabbing them ventrally in the thorax with a minutin pin contaminated with the heat killed pathogen solution. All aphids were born within 24 h of one another to reduce differences among individuals. We allowed aphids to heal in a clean dish before we put them individually onto plants. We monitored survival and counted their offspring every 2–4 days. We removed offspring from plants after counting to prevent overcrowding, and trimmed the plants as necessary.

We conducted two experiments. In Experiment 1, we used heat-killed solutions of the bacterial pathogens *Enterobacter* Ng5b (Gram −) and *Bacillus* n1324b (Gram +) and the aphid-specific fungal pathogen *Z. occidentalis*. We also included two control conditions by stabbing aphids with sterile Ringers solution and by handling unstabbed aphids. We blocked Experiment 1 into two replicates, and used aphid genotype 5A0 (66 aphids per treatment). In Experiment 2, to extend our experiment to additional pathogen species and an additional aphid genotype, we stabbed aphids with either sterile Ringers solution, a solution of heat-killed bacterial pathogen *Serratia* s8d (Gram −), or a solution of heat killed *P. neoaphidis,* an aphid specific fungal pathogen. We used two genotypes (LSR1-01 and 5A0) and included 56 individuals per treatment per genotype.

### Statistical methods

To confirm bacterial virulence, we used survival analysis, fitting a non-parametric (cox proportional hazard) model to analyze survival after confirming that the assumption of proportional hazards was met. We conducted a post-hoc multiple comparisons test to determine which levels were significantly different within bacterial treatment, using the “multcomp” package in R.

For assays of costs to pathogen signal exposure, for Experiment 1 we analyzed total reproduction, last day of reproduction, and day of death using analyses of variance (ANOVA), followed by Tukey's HSD *post hoc* tests, after Yeo-Johnson power transformations (lambda = 3.164, 1.964, 1.621 respectively, using the “car” package in R) to correct for deviation from assumptions of normality and homogeneity of variance. We analyzed total reproduction from Experiment 2 in the same fashion (lambda = 2.306). In both experiments we excluded individuals that had fewer than 10 offspring or that died within the first 6 days, as these were likely damaged from the experimental exposure. We used R (2.10.0, R Development Core Team, 2010) for all analyses.

## Results

### Confirming pathogen virulence

Both fungal pathogens, *Z. occidentalis* and *P. neopahidis* significantly reduce aphid survival upon infection (Ferrari et al. 2001; Parker et al. 2013). Exposure to each of the three bacterial strains used here also significantly reduced aphid survival (Figure S1). In both infection assays, bacterial treatment significantly reduced survival (Infection 1 – ng5b and s8d, *χ*^2^ = 40.51, 2 df, *P* < 0.0001; Infection 2 – ng5b and n1324b, *χ*^2^ = 13.67, 2 df, *P *=* *0.001). The Gram-negative *Enterobacter* bacterium ng5b was the most virulent, killing aphids significantly faster than the Gram-negative bacterium *Serratia* s8d (*z* = 3.25, *P *=* *0.003) and the Gram-positive bacterium *Bacillus* n1324b (*z* = 2.34, *P *=* *0.048).

### Costs of pathogen signal exposure

#### Experiment 1

**Figure 1:**
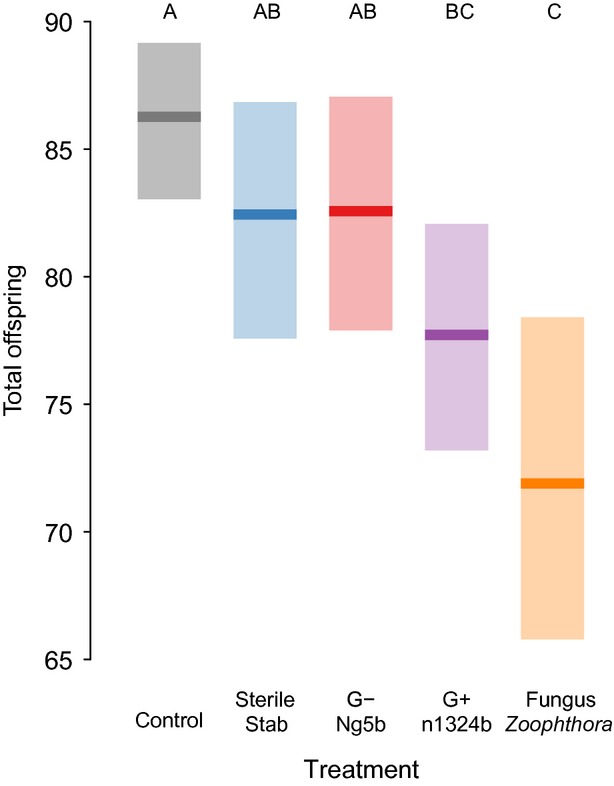
Mean lifetime reproduction ± bootstrapped 95% confidence intervals for naïve control aphids (clone 5A0) or aphids stabbed with a needle dipped in either sterile saline, heat-killed Gram-negative, Gram-positive or fungal challenge (Experiment 1). Letters denote Tukey's HSD groups.

Exposure to heat-killed pathogens significantly influenced lifetime reproduction (Figure 1; *F*_4, 282 _= 5.91, *P *<* *0.001) with aphids exposed to the entomopathogenic fungus *Z. occidentalis* having lower reproduction than any other exposure group, although it was statistically indistinguishable from aphids exposed to the Gram-positive bacteria n1324b. Exposure did not significantly influence the length of reproductive period or longevity (Figs. S2, S3, *F*_4, 282 _= 2.10, *P *=* *0.08; *F*_4, 282 _= 1.85, *P *=* *0.12, respectively). Block had a significant effect on total reproduction (*F*_1,282_ = 11.69, *P* = 0.0008), but there was no significant interaction between replicate block and exposure indicating that the treatment effects were consistent across blocks.

#### Experiment 2

**Figure 2:**
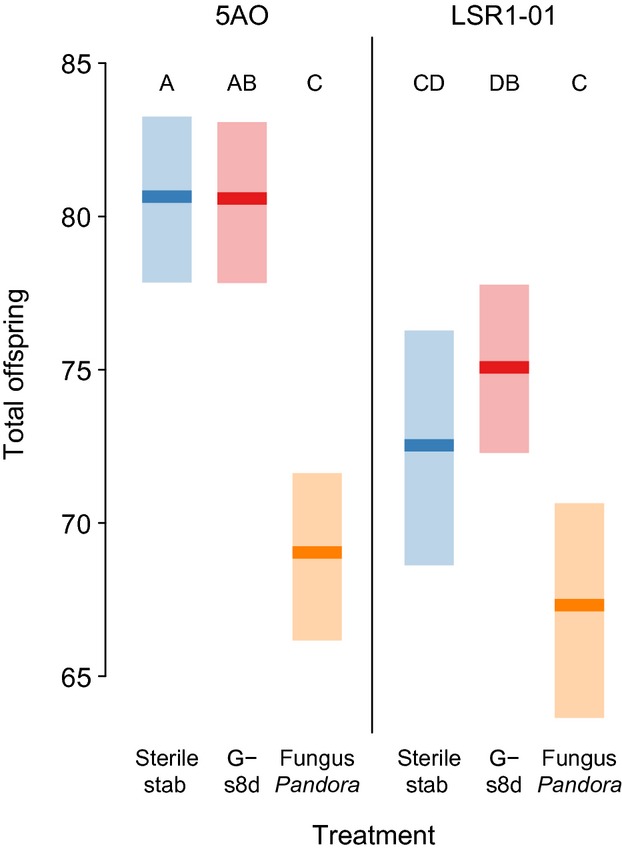
Mean lifetime reproduction ± bootstrapped 95% confidence intervals for aphids from two clones (5A0, LSR1-01) stabbed with a needle dipped in either sterile saline, heat-killed Gram-negative or fungal challenge (Experiment 2). Letters denote Tukey's HSD groups.

Aphids of two genotypes given the second suite of heat-killed pathogens again had significantly reduced lifetime reproduction (Fig. 2, *F*_2, 302 _= 25.26, *P *<* *0.0001) with exposure to the fungal pathogen *P. neoaphidis* reducing fecundity. Exposure to the Gram-negative bacteria s8d did not significantly alter reproduction. The genotypes differed in overall fecundity, but there was no significant interaction between aphid genotype and treatment.

## Discussion

Aphids suffered fitness costs after exposure to signals of two ecologically relevant fungal pathogens. There was no difference between sterile-stab and unstabbed control aphids, indicating that the fitness loss was the result of pathogen exposure not of wounding. Exposure to either of two different heat-killed Gram-negative bacteria, one highly and one moderately virulent (Fig. S1), failed to significantly reduce any fitness measures. Increasing bacterial concentration (Experiment 2) also did reduce fitness. Exposure to the Gram-positive antigen did slightly reduce reproduction, though there was no significant reduction compared to sterile stab controls. Furthermore, there was no aphid genotype by treatment interaction, indicating that the response to fungal elicitors and insensitivity to bacterial elicitors was consistent across these genotypes, which are genetically distinct and collected from different populations. Further sampling could, of course, reveal variation in these responses, which would be a starting point to investigate the ecological pressures mediating immune system evolution. These results are consistent with previous studies that did not detect substantial immune responses to bacterial challenge using transcriptomic (Altincicek et al. 2008; Gerardo et al. 2010), proteomic (Gerardo et al. 2010) and immunological assays (Altincicek et al. 2008, 2011; Laughton et al. 2011).

Fitness costs of an immune response can therefore be detected in pea aphids, but these costs are only apparent when aphids are given fungal cues. Pea aphids lack most of the IMD pathway and many of the effector molecules presumed necessary to deal with infection (Gerardo et al. 2010). In this way we use the pea aphid immune system as a natural knockout, as it lacks one arm of the insect immune response. The specificity of insect humoral pathways is poorly understood (Dionne and Schneider 2008), but the IMD pathway is critical for fighting many Gram-negative bacteria in *Drosophila* (Lemaitre et al. 1997), and studies have found changes in susceptibility to some fungal pathogens in IMD knock-out *Drosophila* (Dionne and Schneider 2008). It has been hypothesized that the loss of the majority of the IMD pathway in pea aphids may be due to their intimate relationship with both intracellular and extracellular Gram-negative bacterial symbionts (Altincicek et al. 2008; Gerardo et al. 2010). As these symbionts are essential for aphid survival, immune activity against them could be detrimental. All aphids have intracellular bacterial symbionts and many other insect species also have relationships with bacteria, but whether other insects share the dramatically diminished immune repertoire of pea aphids remains to be seen. The rapidly expanding collection of insect full genome sequences will help to clarify the flexibility in the organization of the insect immune system.

Pea aphids do, however, retain other important pathways (i.e., Toll, JNK and JAK/STAT pathways) and can phagocytose invading microbes (Laughton et al. 2011; Schmitz et al. 2012). These mechanisms may underlie responses that led to slightly decreased fecundity after exposure to Gram-positive bacterial elicitors and significantly decreased fecundity after exposure to fungal elicitors. However, unknown mechanisms may be at play as well, particularly given that insights gained into insect immune gene repertoires through genomics are revealing surprising deviations from the presumed canonical immune gene set (Evans et al. 2006). Our results suggest that further characterization of the mechanisms of aphid immune responses should utilize a variety of pathogens, and, in particular, include fungi.

In general, when studying the immune system of a non-model host, it is not clear what parasite challenge one should study, and how to measure a fitness response to parasite challenge (Boughton et al. 2011). This work suggests that measuring life-history traits after pathogen exposure provides valuable information about natural host-pathogen interactions in non-model systems. Specifically, our finding that aphids respond to fungus through a costly response opens the door for investigations into the molecular mechanisms behind aphid-fungal resistance. Our findings will also facilitate study of the evolution of aphid-fungal pathogen interactions and how these interactions are shaped by the coupling of host immune responses and aphid bacterial symbionts known to confer protection against fungal pathogens (Scarborough et al. 2005; Parker et al. 2013).

## Supporting Information

**Figure S1**. Survival curves from live bacterial virulence trials. **Figure S2**. Proportion of aphids reproducing over time after challenge with heat killed pathogens or sterile stab. **Figure S3**. Proportion of aphids reproducing over time after challenge with heat killed pathogens or sterile stab.
