# Supplementary material for: Exposure to natural pathogens reveals costly aphid response to fungi but not bacteria
Source: Ecol Evol. 2014 Jan 23;4(4):488–93. doi: 10.1002/ece3.892 (PMC3936394; doi:10.1002/ece3.892)
Supplement: Supplementary file 1 — Figure S1. Survival curves from live bacterial virulence trials. Figure S2. Proportion of aphids reproducing over time after challenge with heat killed pathogens or sterile stab. Figure S3. Proportion of aphids reproducing over time after challenge with heat killed pathogens or sterile stab. [file ece30004-0488-sd1.docx]

**Supplementary Information**

**Figure S1. Survival curves from live bacterial virulence trials.** The survival of aphids stabbed with a minutin pin dipped in sterile LB (Control) or live bacterial solution was monitored for five days. Two trials were conducted, **(Left):** comparing the two Gram negative bacteria (s8d and ng5b) and **(Right):** comparing the Gram positive bacteria (n1324b) with ng5b.

**Figure S2. Proportion of aphids reproducing over time after challenge with heat killed pathogens or sterile stab.**

**Figure S3. Proportion of aphids alive after challenge with heat killed bacteria or sterile stabs.**

1 Hothorn, T., Bretz, F. & Westfall, P. 2008 Simultaneous inference in general parametric models. *Biom J* **50**, 346–363. (doi:10.1002/bimj.200810425)
